# Spoligotype‐based population structure of *Mycobacterium tuberculosis* in the Jimma Zone, southwest Ethiopia

**DOI:** 10.1002/mbo3.744

**Published:** 2018-10-15

**Authors:** Gemeda Abebe, Ketema Abdissa, Kedir Abdella, Mulualem Tadesse, Adane Worku, Gobena Ameni

**Affiliations:** ^1^ Mycobacteriology Research Center Jimma University Jimma Ethiopia; ^2^ Aklilu Lemma Institute of Pathobiology Addis Ababa University Addis Ababa Ethiopia

**Keywords:** Ethiopia, Jimma, *M. tuberculosis*, populations structure

## Abstract

**Background:**

To understand the population dynamics and propose more effective preventive strategies, defining the population structure of the circulating *Mycobacterium tuberculosis* strains is important.

**Methods:**

A total of 177 *M. tuberculosis* complex isolates from pulmonary tuberculosis (TB) cases in southwest Ethiopia were genotyped by spoligotyping. Of the strains included in this study, 126 were pan‐susceptible strains while the remaining 51 isolates were resistant to one or more first‐line anti‐TB drugs. The genotyping results were compared to the international spoligotyping (SITVIT) database of the Pasteur Institute of Guadeloupe and the newly revised publicly available international multi‐marker database (SITVITWEB/SPOLDB4). An online tool Run TB‐Lineage was also used to predict the major lineages using a conformal Bayesian network analysis.

**Results:**

The spoligotyping of the 177 isolates resulted in 69 different spoligotype patterns of which 127 (71.8%) were clustered into 19 spoligoclusters (with clustering rate of 61.02%). Each cluster contains 2–29 isolates. Of the isolates with corresponding SIT in SITVIT/SDB4, the predominant strains identified were SIT 37 of the T3 subfamily with 29 isolates followed by SIT 53 of the T1 subfamily with 20 isolates. SIT 777 of the H4 subfamily and SIT 25 of the CAS1_DELHI subfamily each consisting of six isolates were identified. Eighty spoligotype patterns were orphan as they were not recorded in the SITVIT2/SPDB4 database. Further classification of the isolates on the basis of major lineages showed that 82.5% and 14.1% of the isolates belonged to Euro‐American and East African Indian lineages, respectively, while 2.8% of the isolates belonged to *Mycobacterium africanum* and 0.6% to Indo‐Oceanic.

**Conclusion:**

The ill‐defined T and H clades were predominant around Jimma. The substantial number of orphans recorded in the study area warrants for additional studies with genotyping methods with better resolution and covering whole areas of southwest Ethiopia.

## Abstract 2

The present study has shown the dominance of ill‐defined T and H clades in the study area. Moreover, a substantial number of isolates were Orphan warranting for additional studies covering the whole geographic area of the southwest Ethiopia and genotypic methods with better resolution.

## 1 BACKGROUND

Tuberculosis (TB) remains a public health challenge globally in spite of the availability of vaccine and drugs for treatment. WHO estimates that currently one third of the world's population is infected with bacteria of the *Mycobacterium tuberculosis* complex, and 10 million cases of active TB disease occur each year, resulting in almost 2 million deaths annually. Ethiopia is one of the 22 high burden countries in terms of the number of TB. In 2016, the estimated TB incidence was 177/100,000 population. The country is also one of the high burden countries for the multidrug‐resistant tuberculosis (MDR‐TB) and HIV (World Health Organization, 2017).

Ethiopia succeeded in reducing the burden of TB over the years. However, there is an increasing trend in drug‐resistant TB. According to WHO 2011 report, drug‐resistant TB was 1.6% among new cases and 12% among retreatment cases (World Health Organization, 2011). In 2017 report of the WHO, the rate increased to 2.7% for new cases and 14% for retreatment cases (World Health Organization, 2017).

Spoligotyping was developed as a genotyping tool to provide information on the structure of the direct region in individual *M. tuberculosis* strains and in the different members of the *M. tuberculosis* complex (Kremer et al., 1999). It is a valuable tool to study the population structure and molecular epidemiology of *M. tuberculosis*. Understanding the genetic diversity of the circulating *M. tuberculosis* strains is important to define the population dynamics and to propose more effective preventive and control strategies. Certain genotypes of *M. tuberculosis* were associated with drug resistance (Glynn, Whiteley, Bifani, Kremer, & Soolingen, 2002; Haeili et al., 2013; Pang et al., 2012).

While the genetic diversity of *M. tuberculosis* lineages in Ethiopia has been investigated (Diriba, Berkessa, Mamo, Tedla, & Ameni, 2013; Garedew et al., 2013), there are little or no data in the southwest part of Ethiopia in general and Jimma area in particular. The objective of this study was to investigate the population structure of the mycobacterial isolates collected during November 2010 to June 2012 from Jimma area.

## 2 METHODS

### 2.1 Source of isolates

The isolates for this study were recovered from the TB patients who were included in the drug resistance survey around Jimma among new and retreatment cases (Abdella, Abdissa, Kebede, & Abebe, 2015; Abebe et al., 2012). The stored isolates were subcultured, and 179 isolates were recovered (115 from the study among new cases (Abebe et al., 2012) and 64 from the study on retreatment cases (Abdella et al., 2015)). They were inactivated at 80°C for 1 hr and transported to Aklilu Lemma Institute of Pathobiology, Addis Ababa University, for spoligotyping using 43 spacer version. The drug resistance patterns of the isolates were previously reported (Abdella et al., 2015; Abebe et al., 2012). Of the isolates included in this study, 126 were pan‐susceptible to the first‐line anti‐TB drugs. The remaining 51 isolates were resistant to one or more first‐line anti‐TB drugs. Fifteen isolates were MDR. The isolates that were not MDR included four rifampicin mono‐resistant, 22 isoniazid‐resistant, 11 resistant against ethambutol, and 16 resistant to streptomycin. Of the 15 MDR‐TB isolates, 11 were also resistant against ethambutol and 14 were resistant against streptomycin.

### 2.2 HIV testing

HIV testing is provided for every presumptive TB case at health facility in Ethiopia. The results of the test were recorded from patients’ cards after having consent.

### 2.3 Spoligotyping

Spoligotyping was performed at the Aklilu Lemma Institute of Pathobiology, Addis Ababa University. One hundred and seventy‐seven (177) isolates confirmed as *M. tuberculosis* using para nitro benzoic acid inhibition test, and RD9 typing was further analyzed by spoligotyping according to the standardized protocol (Kamerbeek et al., 1997) and following manufacturer's instructions (reagents from Ocimum Biosolution, custom Master Mix from ABgene). Two (2) isolates DNA were not amplified and therefore not spoligotyped. The presence of spacers was visualized on film as black squares after incubation with streptavidin‐peroxidase and enhanced chemiluminescence detection reagents (RPN 2105 Amersham, GE Healthcare Bio‐Sciences). The spacer hybridization was read by two independent readers; in case of discrepancy, the opinion of a third reader was considered. The patterns were translated into binary and octal format as previously described (Dale et al., 2001). The 43‐digit binary code was converted to 15‐digit octal code (base 8, having the digits 0–7).

### 2.4 Genotype database comparison and analysis

The spoligotyping results were compared to the international spoligotyping (SITVIT) database of the Pasteur Institute of Guadeloupe (https://www.pasteur-guadeloupe.fr:8081/SITVITDemo) (Brudey et al., 2006) and the newly revised publicly available international multimarker database (SITVITWEB) (Demay et al., 2012). Orphan strains were further compared with MIRU‐VNTR*plus*, an online database (https://www.miru-vntrplus.org) (Weniger, Krawczyk, Supply, Niemann, & Harmsen, 2010). A SIT (spoligotype international type) was assigned to the isolates that share an identical spoligotype pattern in the database, while spoligotype patterns that had not been registered before were defined as “orphan.” Spoligotype patterns that did not cluster with other patient isolates were defined as unique. An online tool Run TB‐Lineage (https://tbinsight.cs.rpi.edu/run_tb_lineage.html) was also used to predict the major lineages using the conformal Bayesian network (CBN) analysis.

### 2.5 Statistical analysis

Statistical analysis was performed using SPSS Statistic 17 (SPSS Inc., USA). Proportions of drug susceptibility test profiles for *M. tuberculosis* strains were compared using chi‐square analysis. The two‐sided Pearson's chi‐square test was used to assess associations of drug resistance profiles with spoligotype families. Furthermore, an association of first‐line anti‐TB drug resistance profiles with MTBC genotypes was estimated and expressed as the odds ratio (OR) and 95% confidence interval (95% CI). A *p* value of <0.05 was considered statistically significant.

### 2.6 Ethics statement

This study was part of the previous studies that were published (Abdella et al., 2015; Abebe et al., 2012). Both studies were approved by ethical review board of College of Health Sciences, Jimma University, and national research ethics review committee (ref. no 3.10/17/03). All patients enrolled in these clinical studies had provided informed consent for further characterization of the mycobacterial isolates.

## 3 RESULTS

### 3.1 Characteristics of the patients from which the isolates were collected

In this study, data generated from 177 study subjects were used in the analysis of demographic, clinical, and mycobacteriological data. Most of the study subjects (54.8%) were males and in the age group of 18–28 years (61.0%). Most (82.5%) of the study participants were new TB cases (Table 1).

**Table 1:** Characteristics of the patients from which the isolates were collected from the Jimma Zone, southwest Ethiopia

Characteristics	Category	*N* (%)
Sex	Male	97 (54.8%)
	Female	80 (45.2%)
Age	18–28	108 (61.0)
	29–39	38 (21.5)
	40–50	24 (13.6)
	>50	7 (4.0)
Family history of TB	Yes	20 (11.3)
	No	157 (88.7)
HIV serostatus	Positive	23 (13.0)
	Negative	76 (42.9)
	Unknown	78 (44.1)
History of diabetes	Yes	6 (3.4%)
	No	171 (96.6%)
TB category	Re‐treatment	31 (17.5)
	New	146 (82.5%)

### 3.2 Spoligotype patterns of the *Mycobacterium tuberculosis* strains

The spoligotyping of the 177 isolates resulted in 69 different spoligotype patterns (with clustering rate of 61.02%). Of the isolates, 127 (71.8%) were clustered into 19 spoligoclusters containing 2–29 isolates per cluster. The remaining 50 (28.2%) isolates were unique which means that the isolates did not cluster with other patient isolates in this study. Over half (97 isolates) of the isolates representing 24 spoligotype patterns were shared types. Of these shared types, the predominant strains identified were SIT 37 of the T3 subfamily consisting of 29 isolates followed by SIT 53 of the T1 subfamily with 20 isolates. In addition to the T family, SIT 777 of the H4 subfamily and SIT 25 of the CAS1_DELHI subfamily each with six isolates were identified (Table 2). Eighty spoligotype patterns were orphans that were not recorded in the SITVIT2/SPDB4 database (Table 3). Further classification of the isolates spoligotype patterns using the TB‐insight RUN TB‐lineage revealed that Euro‐American lineages accounted for 82.5%, East African Indian for 14.1%, *Mycobacterium africanum* for 2.8%, and Indo‐Oceanic for 0.6% (Tables 2 and 3).

**Table 2:**
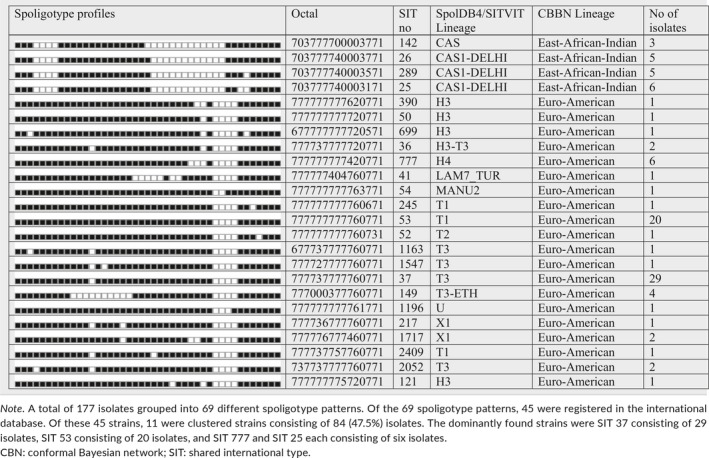
Description of 24 shared types (SITs; *n* = 97 isolates) which have already been registered in the SITVIT2 or SpolDB4 database and corresponding spoligotyping defined lineages/sub‐lineages isolated from the Jimma Zone, southwest Ethiopia

**Table 3:**
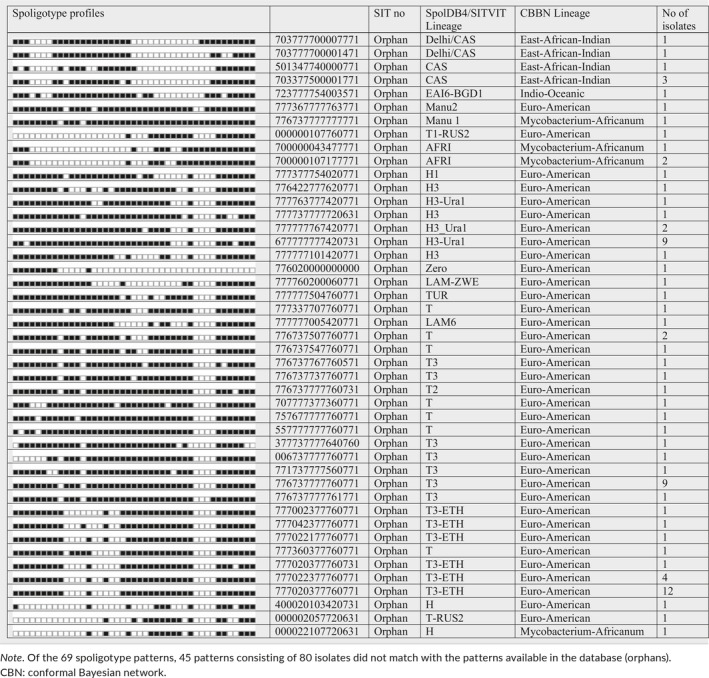
Description of 45 orphan strains (*n* = 80) and corresponding spoligotyping defined lineages/sub‐lineages recorded among *M. tuberculosis* strains isolated from the Jimma Zone, southwest Ethiopia

### 3.3 *M. tuberculosis* drug resistance by lineage and family

The result of drug sensitivity pattern of the isolates is presented in Table 4. Compared with other strains, CAS1_DELHI subfamily was more likely to be resistant to the first‐line ant‐TB drugs and to be MDR‐TB strains (Table 4).

**Table 4:** Comparison of CAS1_DELHI and other clades for drug resistance in southwest Ethiopia

Clade	Resistance %				
	RIF	INH	S	E	MDR
CAS1_DELHI	23.1	34.6	42.3	26.9	19.2
Others	8.6	18.5	12.6	9.9	10.0
OR (95% CI)	3.19 (1.09–9.33)	2.33 (0.94–5.76)	5.1 (2.04–12.72)	3.34 (1.21–9.24)	3.36 (1.05–10.79)
*p* Value	0.04	0.07	0.0001	0.02	0.04

Note

E: ethambutol; INH: isoniazid; MDR: multidrug resistant; RIF: rifampicin; S: streptomycin.

## 4 DISCUSSION

In the present study, the population structure of *M. tuberculosis* isolates was investigated in adults around Jimma, southwest Ethiopia. Our previous report from this area indicated the prevalence of pulmonary TB was lower compared to the national prevalence for Ethiopia (Deribew et al., 2012). The current report suggests that there is high genetic diversity of *M. tuberculosis* around Jimma. Previous reports have also indicated that in areas with low prevalence of TB high genetic diversity is expected (Lopez‐Avalos et al., 2017; Michel et al., 2008) since the dominance and transmission of a single strain is less likely, rather TB in such settings is the result of reactivation from previous infections. This is substantiated in the current study by the fact that there were 69 spoligotype patterns from the 177 *M. tuberculosis* isolates typed.

On the other hand, our study suggests that the genetic diversity of *M. tuberculosis* in southwest part of Ethiopia is not well studied as there were a substantial number of “Orphan” strains lacking corresponding patterns from the available international databases. It is known that spoligotyping has relatively low resolution power in terms of differentiation of clusters, thereby indicating strain differences. Thus, to make more understanding of the *M. tuberculosis* strains in the study area, further study with other methods having better resolution power is recommended.

Majority of the isolates with SITs reported in this study (SIT 37 and SIT 53) were dominated by modern clades of the ill‐defined T and H families that were consistently reported from Ethiopia (Bedewi et al., 2017; Belay et al., 2013; Getahun et al., 2015; Maru, Mariam, Airgecho, Gadissa, & Aseffa, 2015). These clades were reported from the study on children in the same study area suggesting their transmission from adults (Workalemahu et al., 2013). This result suggests that these specific families may have shown successful epidemiological fitness in this geographic area. The ancient clades were also reported in this study with CAS family and Manu being the main clades. It is in agreement with the previous reports that TB originated at the beginning from Ethiopia with the ancient clades and the modern clades also imported following human population movement (Comas et al., 2015). The T3_ETH is the most dominant strain circulating in Ethiopia. Though these strains are new to the database/orphans, they are reported in southwest and other regions of Ethiopia (Tadesse et al., 2017; Tessema et al., 2013; Zewdie et al., 2016).

This study suggests that CAS1_DELHI clades were more likely to be associated with resistance to first‐line anti‐TB drugs. In agreement with our finding, a study from New Delhi has also concluded that CAS1_DELHI isolates have a high frequency of mutations in the *rpoB* and* katG* genes which were indicators for resistance against rifampicin and isoniazid, respectively (Stavrum, Myneedu, Arora, Ahmed, & Grewal, 2009).

In this study, a substantial number (28.2%) of isolates have exhibited unique spoligotype pattern suggesting that these cases could be the result of reactivation from past infection (Murray, 2002). It is very well understood that in Ethiopia, the rate of infection with *M. tuberculosis* is high. However, for the clinical form of TB to be observed, the immune status of the infected cases and other internal biological factors must support the progression. By the time that the isolates were collected, the rate of HIV infection was relatively high supporting the idea of reactivation TB (Fedral HIV/AIDS Prevention and Control Office of Ethiopia, 2014).

Our study is not without limitations. We used spoligotyping which does have less discriminatory power in identifying the different clades. The patterns indicated to be similar by spoligotyping could be different, thus cannot be used to estimate transmission dynamics. Moreover, the small number of isolates typed in the present study could present a potential bias regarding representativeness of the strains of MTBC circulating around Jimma.

In conclusion, the present study has shown the dominance of ill‐defined T and H clades in the study area. Moreover, a substantial number of isolates were Orphan warranting for additional studies covering the whole geographic area of the southwest Ethiopia and genotyping methods with better resolution.

## CONFLICT OF INTEREST

We authors declare that no competing interests exist.

## AUTHORS CONTRIBUTION

GAbebe conceived and designed the study, was involved in data collection, cleaned data, and wrote the initial draft of the paper. KAbdissa, KAbdella, and MT were responsible for culturing and DST analysis of the isolates. AW and GAmeni did spoligotyping. All authors reviewed and gave input to the subsequent manuscript drafts.

## Data Availability

All data are provided in full in the results section of this paper.

## References

[mbo3744-bib-0001] Abdella, K. , Abdissa, K. , Kebede, W. , & Abebe, G. (2015). Drug resistance patterns of *Mycobacterium tuberculosis* complex and associated factors among retreatment cases around Jimma, southwest Ethiopia. BMC Public Health, 15(599). 10.1186/s12889-015-1955-3 PMC4489121 26135909

[mbo3744-bib-0002] Abebe, G. , Abdissa, K. , Abdissa, A. , Apers, L. , Agonafir, M. , de‐Jong, B. C. , & Colebunders, R. (2012). Relatively low primary drug resistant tuberculosis in southwestern Ethiopia. BMC Research Notes, 5, 225 10.1186/1756-0500-5-225 22574696 PMC3441821

[mbo3744-bib-0003] Bedewi, Z. , Worku, A. , Mekonnen, Y. , Yimer, G. , Medhin, G. , Mamo, G. , … Ameni, G. (2017). Molecular typing of *Mycobacterium tuberculosis* complex isolated from pulmonary tuberculosis patients in central Ethiopia. BMC Infectious Diseases, 17(1), 184 10.1186/s12879-017-2267-2 28249607 PMC5333391

[mbo3744-bib-0004] Belay, M. , Ameni, G. , Bjune, G. , Couvin, D. , Rastogi, N. , & Abebe, F. (2013). Strain diversity of *Mycobacterium tuberculosis* isolates from pulmonary tuberculosis patients in Afar pastoral region of Ethiopia. BioMed Research International, 2014, 238532. 10.1155/2014/238532 PMC3966356 24734230

[mbo3744-bib-0005] Brudey, K. , Driscoll, J. R. , Rigouts, L. , Prodinger, W. M. , Gori, A. , Al‐Hajoj, S. A. , … Sola, C. (2006). *Mycobacterium tuberculosis* complex genetic diversity: Mining the fourth international spoligotyping database (SpolDB4) for classification, population genetics and epidemiology. BMC Microbiology, 6, 23. 16519816 10.1186/1471-2180-6-23 PMC1468417

[mbo3744-bib-0006] Comas, I. , Hailu, E. , Kiros, T. , Bekele, S. , Mekonnen, W. , Gumi, B. , … Berg, S. (2015). Population genomics of *Mycobacterium tuberculosis* in Ethiopia contradicts the virgin soil hypothesis for human tuberculosis in sub‐Saharan Africa. Current Biology, 25(24), 3260–3266. 10.1016/j.cub.2015.10.061 26687624 PMC4691238

[mbo3744-bib-0007] Dale, J. W. , Brittain, D. , Cataldi, A. A. , Cousins, D. , Crawford, J. T. , Driscoll, J. , … Vincent , V. (2001). Spacer oligonucleotide typing of bacteria of the *Mycobacterium tuberculosis* complex: Recommendations for standardised nomenclature. The International Journal of Tuberculosis and Lung Disease, 5(3), 216–219. 11326819

[mbo3744-bib-0008] Demay, C. , Liens, B. , Burguiere, T. , Hill, V. , Couvin, D. , Millet, J. , … Rastogi, N. (2012). SITVITWEB—A publicly available international multimarker database for studying *Mycobacterium tuberculosis* genetic diversity and molecular epidemiology. Infection, Genetics and Evolution, 12(4), 755–766. 10.1016/j.meegid.2012.02.004 22365971

[mbo3744-bib-0009] Deribew, A. , Abebe, G. , Apers, L. , Abdissa, A. , Deribe, F. , Woldemichael, K. , … Colebunders, R. (2012). Prevalence of pulmonary TB and spoligotype pattern of *Mycobacterium tuberculosis* among TB suspects in a rural community in southwest Ethiopia. BMC Infectious Diseases, 12, 54 10.1186/1471-2334-12-54 22414165 PMC3378444

[mbo3744-bib-0010] Diriba, B. , Berkessa, T. , Mamo, G. , Tedla, Y. , & Ameni, G. (2013). Spoligotyping of multidrug‐resistant *Mycobacterium tuberculosis* isolates in Ethiopia. The International Journal of Tuberculosis and Lung Disease, 17(2), 246–250. 23317962 10.5588/ijtld.12.0195

[mbo3744-bib-0011] Fedral HIV/AIDS Prevention and Control Office of Ethiopia (2014). HIV/AIDS in Ethiopia: An epidemiological synthesis. Addis Ababa, Ethiopia: Fedral HIV/AIDS Prevention and Control Office of Ethiopia.

[mbo3744-bib-0012] Garedew, L. , Mihret, A. , Mamo, G. , Abebe, T. , Firdessa, R. , Bekele, Y. , Ameni, G. (2013). Strain diversity of mycobacteria isolated from pulmonary tuberculosis patients at Debre Birhan Hospital, Ethiopia. The International Journal of Tuberculosis and Lung Disease, 17(8), 1076–1081. 10.5588/ijtld.12.0854 23827032

[mbo3744-bib-0013] Getahun, M. , Ameni, G. , Kebede, A. , Yaregal, Z. , Hailu, E. , Medihn, G. , … Lemma, E. (2015). Molecular typing and drug sensitivity testing of *Mycobacterium tuberculosis* isolated by a community‐based survey in Ethiopia. BMC Public Health, 15, 751 10.1186/s12889-015-2105-7 26245282 PMC4527252

[mbo3744-bib-0014] Glynn, J. R. , Whiteley, J. , Bifani, P. J. , Kremer, K. , & van Soolingen, D. (2002). Worldwide occurrence of Beijing/W strains of *Mycobacterium tuberculosis*: A systematic review. Emerging Infectious Diseases, 8(8), 843–849. 12141971 10.3201/eid0808.020002 PMC2732522

[mbo3744-bib-0015] Haeili, M. , Darban‐Sarokhalil, D. , Fooladi, A. A. , Javadpour, S. , Hashemi, A. , Siavoshi, F. , … Feizabadi , M. M. (2013). Spoligotyping and drug resistance patterns of *Mycobacterium tuberculosis* isolates from five provinces of Iran. MicrobiologyOpen, 2(6), 988–996. 24311556 10.1002/mbo3.139 PMC3892344

[mbo3744-bib-0016] Kamerbeek, J. , Schouls, L. , Kolk, A. , van Agterveld, M. , van Soolingen, D. , Kuijper, S. , … van Embden , J. (1997). Simultaneous detection and strain differentiation of *Mycobacterium tuberculosis* for diagnosis and epidemiology. Journal of Clinical Microbiology, 35(4), 907–914. 9157152 10.1128/jcm.35.4.907-914.1997 PMC229700

[mbo3744-bib-0017] Kremer, K. , van Soolingen, D. , Frothingham, R. , Haas, W. H. , Hermans, P. W. M. , Martín, C. , … van Embden, J. D. (1999). Comparison of methods based on different molecular epidemiological markers for typing of *Mycobacterium tuberculosis* complex strains: Interlaboratory study of discriminatory power and reproducibility. Journal of Clinical Microbiology, 37, 2607–2618. 10405410 10.1128/jcm.37.8.2607-2618.1999 PMC85295

[mbo3744-bib-0018] Lopez‐Avalos, G. , Gonzalez‐Palomar, G. , Lopez‐Rodriguez, M. , Vazquez‐Chacon, C. A. , Mora‐Aguilera, G. , Gonzalez‐Barrios, J. A. , … Alvarez‐Maya , I. (2017). Genetic diversity of *Mycobacterium tuberculosis* and transmission associated with first‐line drug resistance: A first analysis in Jalisco, Mexico. Journal of Global Antimicrobial Resistance, 11, 90–97. 28760681 10.1016/j.jgar.2017.07.004

[mbo3744-bib-0019] Maru, M. , Mariam, S. H. , Airgecho, T. , Gadissa, E. , & Aseffa, A. (2015). Prevalence of tuberculosis, drug susceptibility testing, and genotyping of mycobacterial isolates from pulmonary tuberculosis patients in Dessie, Ethiopia. Tuberculosis Research and Treatment, 2015, 215015. 26180642 10.1155/2015/215015 PMC4477223

[mbo3744-bib-0020] Michel, A. L. , Hlokwe, T. M. , Coetzee, M. L. , Mare, L. , Connoway, L. , Rutten, V. P. , & Kremer, K. (2008). High *Mycobacterium bovis* genetic diversity in a low prevalence setting. Veterinary Microbiology, 126(1–3), 151–159. 10.1016/j.vetmic.2007.07.015 17720336

[mbo3744-bib-0021] Murray, M. (2002). Determinants of cluster distribution in the molecular epidemiology of tuberculosis. Proceedings of the National Academy of Sciences of the United States of America, 99(3), 1538–1543. 10.1073/pnas.022618299 11818527 PMC122226

[mbo3744-bib-0022] Pang, Y. , Zhou, Y. , Zhao, B. , Liu, G. , Jiang, G. , Xia, H. , … Zhao , Y.‐l. (2012). Spoligotyping and drug resistance analysis of *Mycobacterium tuberculosis* strains from national survey in China. PLoS One, 7(3), e32976. 22412962 10.1371/journal.pone.0032976 PMC3296750

[mbo3744-bib-0023] Stavrum, R. , Myneedu, V. P. , Arora, V. K. , Ahmed, N. , & Grewal, H. M. (2009). In‐depth molecular characterization of *Mycobacterium tuberculosis* from New Delhi—Predominance of drug resistant isolates of the 'Modern' (TbD1) type. PLoS One, 4(2), e4540 10.1371/journal.pone.0004540 19234602 PMC2641002

[mbo3744-bib-0024] Tadesse, M. , Abebe, G. , Bekele, A. , Bezabih, M. , de Rijk, P. , Meehan, C. J. , … Rigouts, L. (2017). The predominance of Ethiopian specific *Mycobacterium tuberculosis* families and minimal contribution of *Mycobacterium bovis* in tuberculous lymphadenitis patients in Southwest Ethiopia. Infection, Genetics and Evolution, 55, 251–259. 10.1016/j.meegid.2017.09.016 28919549

[mbo3744-bib-0025] Tessema, B. , Beer, J. , Merker, M. , Emmrich, F. , Sack, U. , Rodloff, A. C. , & Niemann, S. (2013). Molecular epidemiology and transmission dynamics of *Mycobacterium tuberculosis* in Northwest Ethiopia: New phylogenetic lineages found in Northwest Ethiopia. BMC Infectious Diseases, 13, 131 10.1186/1471-2334-13-131 23496968 PMC3605317

[mbo3744-bib-0026] Weniger, T. , Krawczyk, J. , Supply, P. , Niemann, S. , & Harmsen, D. (2010). MIRU‐VNTRplus: A web tool for polyphasic genotyping of *Mycobacterium tuberculosis* complex bacteria. Nucleic Acids Research, 38 (Web Server issue), W326–W331. 10.1093/nar/gkq351 20457747 PMC2896200

[mbo3744-bib-0027] Workalemahu, B. , Berg, S. , Tsegaye, W. , Abdissa, A. , Girma, T. , Abebe, M. , & Aseffa, A. (2013). Genotype diversity of *Mycobacterium* isolates from children in Jimma, Ethiopia. BMC Research Notes, 6, 352 10.1186/1756-0500-6-352 24007374 PMC3766673

[mbo3744-bib-0028] World Health Organization (2011). Global tuberculosis control 2011. Geneva, Switzerland: World Health Organization.

[mbo3744-bib-0029] World Health Organization (2017). Global tuberculosis report 2017. Geneva, Switzerland: World Health Organization.

[mbo3744-bib-0030] Zewdie, O. , Mihret, A. , Ameni, G. , Worku, A. , Gemechu, T. , & Abebe, T. (2016). Molecular typing of mycobacteria isolated from tuberculous lymphadenitis cases in Ababa, Ethiopia. The International Journal of Tuberculosis and Lung Disease, 20(11), 1529–1534. 10.5588/ijtld.15.1023 27776596 PMC5665165

